# Production of Recombinant Human Ceruloplasmin: Improvements and Perspectives

**DOI:** 10.3390/ijms22158228

**Published:** 2021-07-30

**Authors:** Maria Carmela Bonaccorsi di Patti, Antimo Cutone, Marek Nemčovič, Zuzana Pakanová, Peter Baráth, Giovanni Musci

**Affiliations:** 1 Department of Biochemical Sciences ‘A. Rossi Fanelli’, Sapienza University of Rome, 00185 Rome, Italy; 2 Department Biosciences and Territory, University of Molise, 86090 Pesche, Italy; antimo.cutone@unimol.it; 3 Institute of Chemistry, Slovak Academy of Sciences, 84538 Bratislava, Slovakia; marek.nemcovic@savba.sk (M.N.); zuzana.pakanova@savba.sk (Z.P.); peter.barath@savba.sk (P.B.)

**Keywords:** ceruloplasmin, ferroxidase, *Pichia pastoris*, glycoengineered yeast, iron, copper, aceruloplasminemia

## Abstract

The ferroxidase ceruloplasmin (CP) plays a crucial role in iron homeostasis in vertebrates together with the iron exporter ferroportin. Mutations in the CP gene give rise to aceruloplasminemia, a rare neurodegenerative disease for which no cure is available. Many aspects of the (patho)physiology of CP are still unclear and would benefit from the availability of recombinant protein for structural and functional studies. Furthermore, recombinant CP could be evaluated for enzyme replacement therapy for the treatment of aceruloplasminemia. We report the production and preliminary characterization of high-quality recombinant human CP in glycoengineered *Pichia pastoris* SuperMan5. A modified yeast strain lacking the endogenous ferroxidase has been generated and employed as host for heterologous expression of the secreted isoform of human CP. Highly pure biologically active protein has been obtained by an improved two-step purification procedure. Glycan analysis indicates that predominant glycoforms HexNAc2Hex8 and HexNAc2Hex11 are found at Asn119, Asn378, and Asn743, three of the canonical four N-glycosylation sites of human CP. The availability of high-quality recombinant human CP represents a significant advancement in the field of CP biology. However, productivity needs to be increased and further careful glycoengineering of the SM5 strain is mandatory in order to evaluate the possible therapeutic use of the recombinant protein for enzyme replacement therapy of aceruloplasminemia patients.

## 1. Introduction

The ferroxidase ceruloplasmin (CP) belongs to the family of the blue multicopper oxidases, characterized by the presence of multiple copper ions, which safely couple the oxidation of substrates to the controlled reduction of oxygen to water without release of potentially toxic intermediates [1,2]. CP is a large multi-domain glycoprotein made up of six cupredoxin domains arranged with a ternary pseudosymmetry. The interface between domains 1 and 6 hosts the catalytically essential trinuclear copper cluster formed by type 2 and type 3 binuclear copper where oxygen is reduced to water. Domains 2, 4, and 6 each harbor a type 1 copper site, where electrons are accepted from the substrate. Two isoforms of CP are found in mammals: secreted CP is mainly synthesized by hepatocytes and released into the plasma, while a GPI-anchored form (CP-GPI) produced by alternative splicing has been identified prevalently in the brain but also in other cell types [3]. Several functions have been attributed to CP, ranging from copper transport to ferrous iron and biological amines oxidation, as well as antioxidant activity via prevention of the formation of free radicals through different mechanisms, but it is now widely accepted that the main physiological role of CP is related to its ferroxidase activity [3,4]. Export of ferrous iron from cells is mediated by ferroportin, the only known mammalian iron exporter; iron is then oxidized by a ferroxidase to facilitate safe loading onto transferrin for distribution throughout the body [5,6,7]. Depending on the cell type, the ferroxidase activity is provided either by CP (soluble or GPI-linked) or by hephaestin, a multicopper oxidase involved in intestinal iron absorption [8] and iron efflux in neurons [9]. The connection between ferroxidases and ferroportin is further strengthened by the finding that the ferroxidase activity of CP or of hephaestin is required for the stability of cell surface ferroportin and the transporter is rapidly internalized and degraded in the absence of the ferroxidase [10,11].

Aceruloplasminemia is a rare genetic disease caused by mutations in the gene of CP [12,13]. Approximately 70 mutations of the CP gene have been described mainly in Japanese and Caucasian patients [14,15,16]. Patients develop retinal degeneration, diabetes mellitus, and neurologic symptoms, which include ataxia, involuntary movements, parkinsonism, mood and behavior disturbances, and cognitive impairment [17]. Characterization of aceruloplasminemia missense mutants has indicated that most mutants lacked ferroxidase activity either due to retention in the endoplasmic reticulum (ER) or to production as apo-CP lacking copper, owing to folding defects [14,18,19]. However, other mutants were partly or fully functional [20,21], indicating that our understanding of the pathogenesis of aceruloplasminemia is still incomplete. Currently, no efficient therapy is available for this disease, but recent data suggest that enzyme replacement therapy could be a valid option for the treatment of aceruloplasminemia [22,23].

Recombinant production of human CP would allow to exploit the power of site-directed mutagenesis to clarify the molecular bases of functional impairment of aceruloplasminemia missense mutants, and it could also provide an answer to many open questions regarding the precise details of the catalytic mechanism of CP and of its interaction with ferroportin. Furthermore, recombinant human CP would represent a virtually unlimited source of protein available to test the validity of enzyme replacement therapy for treatment of aceruloplasminemia patients. Despite the fact that the coding sequence of human CP has been known since 1986 [24], a survey of the literature shows that, apart from our previous work on aceruloplasminemia mutants [20,25], only one paper published in 2001 reports expression and purification of limited amounts of recombinant human CP, using the yeast *Pichia pastoris* as host [26]. All other papers dealing with the characterization of CP mutants employ transient transfection of mammalian cell lines for functional analyses and no purification of the recombinant protein is performed (e.g., [21,27,28]).

Here, we report production and preliminary characterization of high-quality recombinant human CP in glycoengineered *Pichia pastoris*. A modified yeast strain lacking the endogenous ferroxidase has been generated and employed as host for heterologous expression of the secreted isoform of human CP. Together with an improved purification procedure, this approach has allowed to obtain a biologically active protein with more homogeneous glycans.

## 2. Results and Discussion

Production of recombinant CP is extremely challenging due to the complexity of the protein: high M_r_ (1046 amino acids for the mature secreted protein isoform), requirement for 6 copper atoms: three T1 plus a T2–T3 trinuclear cluster for enzymatic activity, N-glycosylation on multiple Asn residues, five disulfide bridges, sensitivity to proteases. We have previously reported the expression of secreted human CP in the yeast *P. pastoris*. However, the protein was only partially purified and it was heavily hyperglycosylated [20,25].

Innovations to overcome the limitations of our former expression system take advantage of the development of new yeast strains and of improved components to tackle some of the problems associated with expression of secreted heterologous proteins. In particular, glycoengineered yeast strains afford control over glycan structure allowing the production of human-like N-glycans. This is a significant improvement if use of the protein for therapeutic purposes is taken into consideration.

### 2.1. Expression of Recombinant CP

**Figure 1:**
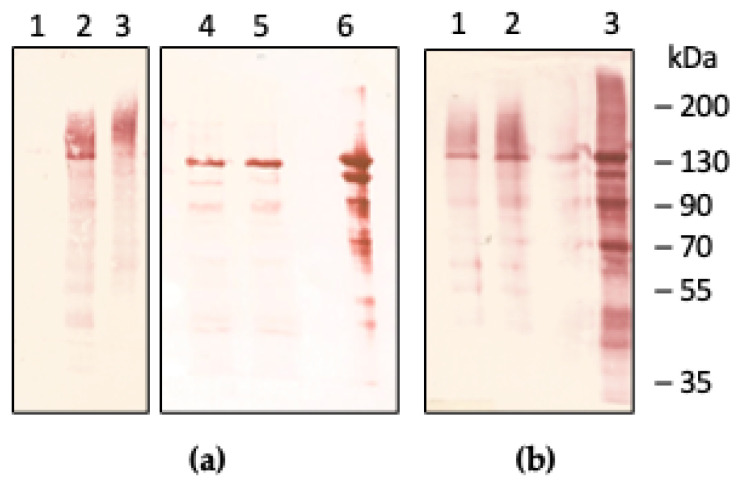
Western blot analysis of expression of recombinant human CP in *P. pastoris*. Panel (**a**): untransformed SM5 *fet3*Δ (lane 1), CP expressed in *fet3*Δ strains derived from SM5 (lane 2) or JC300 (lane 3); samples after treatment with EndoH are shown in lanes 4 (SM5) and 5 (JC300); lane 6, plasma-derived CP (200 ng). Panel (**b**): pOst1-alfaF-CP (lane 1) and CP with its native signal peptide (lane 2) expressed in SM5 *fet3*Δ. Lane 3, plasma-derived CP (200 ng). Yeast cells were grown to OD_600_ = 2 and culture supernatants were collected and concentrated 30-fold; 20 μL of concentrated culture supernatants was loaded on the gel.

Heterologous expression of the secreted isoform of human CP under control of the constitutive GAP promoter has been performed in the *P. pastoris* SM5 strain. This strain is engineered to produce GlcNAc2Man5 glycans by way of inactivation of the gene of the α-1,6-mannosyltransferase Och1, and by constitutive overexpression of an ER-retained HDEL-tagged *T. reesei* α-1,2-mannosidase [29,30]. Inactivation of OCH1 prevents addition of mannose residues on the GlcNAc2Man8 core, which is trimmed to GlcNAc2Man5 by *T. reesei* mannosidase [29]. Inactivation of the gene for the ferroxidase FET3 by the split marker strategy has been achieved in the SM5 strain, in order to avoid any possible background contamination with endogenous ferroxidase activity during expression of CP. Two mutations have been introduced in the coding sequence of CP to replace Arg481 and Lys887 with Gln to remove the two major sites responsible for the sensitivity of CP to proteolysis [31] (residue numbering is of the mature protein without the signal peptide). Furthermore, in the effort to improve secretion levels of the recombinant protein the native signal sequence of human CP has been replaced with a hybrid signal sequence derived from *S. cerevisiae* Ost1 and pro-alfa-factor, which directs co-translational translocation across the ER membrane [32]. Western blot analysis of culture supernatants from *P. pastoris fet3*Δ strains expressing recombinant human CP either with the native or the modified signal sequence is shown in Figure 1.

Compared to JC300, expression levels of CP in glycoengineered SM5 were similar but a much more homogeneous protein with a band at the same M_r_ of plasma-derived CP and a component with higher M_r_ was produced (Figure 1a). Treatment with EndoH yielded a band with M_r_ close to 120 kDa, indicating that the presence of N-glycans was responsible for the higher M_r_ component of CP. The yield of recombinant CP was not improved by replacement of the native signal sequence with the hybrid signal derived from Ost1 and the pro-alfa-factor sequences [32] (Figure 1b). This finding suggests that co-translational translocation of the nascent polypeptide chain to the ER lumen is effective with the native signal sequence of CP, confirming the ability of *P. pastoris* to correctly recognize and process mammalian signal peptides.

### 2.2. Purification and Characterization of Recombinant CP

**Figure 2:**
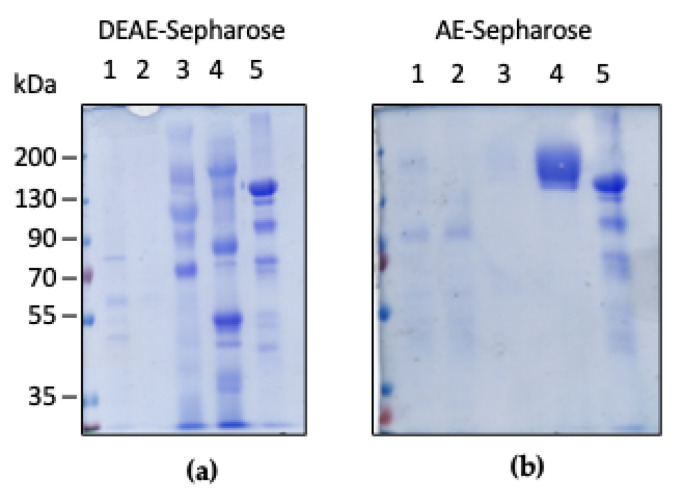
SDS-PAGE analysis of recombinant CP purified on DEAE-Sepharose (**a**) and AE-Sepharose (**b**). Panel (**a**): lane 1, input; lane 2, unbound fraction; lane 3, fraction eluted with NaCl 150 mM; lane 4, fraction eluted with NaCl 250 mM; lane 5, plasma-derived CP (5 μg). Panel (**b**): lane 1, input; lane 2, unbound fraction; lane 3, fraction eluted with NaCl 250 mM; lane 4, fraction eluted with NaCl 500 mM (8 μg); lane 5, plasma-derived CP (5 μg).

Human CP with the native signal sequence has been produced in the SM5 *fet3*Δ strain grown in minimal medium supplemented with CuSO_4_ and Fe(NH_4_)_2_(SO_4_)_2_. Highly pure recombinant CP has been obtained by a two-step chromatography procedure (Figure 2).

A first anion exchange chromatography of culture supernatants on DEAE-Sepharose yielded a fraction eluted at 150 mM NaCl that was enriched in CP (Figure 2a). This fraction was further purified on AE-Sepharose, a chloroethylamine-derivatized Sepharose matrix with high affinity for CP [33,34]. At variance with the hyperglycosylated recombinant protein produced in our previous work [20,25], CP bound strongly to AE-Sepharose and it was eluted at 500 mM NaCl. SDS-PAGE analysis demonstrated that the protein had a high degree of purity, and no degradation was apparent. The recombinant CP exhibited a slightly higher M_r_ compared to the plasma-derived protein (Figure 2b, lane 4 vs. lane 5). The yield of pure recombinant human CP was about 0.1 mg from 1 L culture.

The enzymatic activity of purified recombinant human CP was assayed with *o*-dianisidine (oDA, a non-physiologic aromatic amine widely used to measure CP enzymatic activity) and iron as substrates. The specific oxidase activity towards oDA and the ferroxidase activity of recombinant CP was 90 and 96%, respectively, of the plasma-derived CP, indicating that the recombinant protein was fully active.

This result confirms that copper incorporation into recombinant CP has taken place correctly and that the yeast ATPase Ccc2, which delivers copper in the late Golgi or post-Golgi compartment [25,35], is able to provide copper during biosynthesis of CP in *P. pastoris* SM5.

### 2.3. Glycan Analysis of Recombinant CP

**Figure 3:**
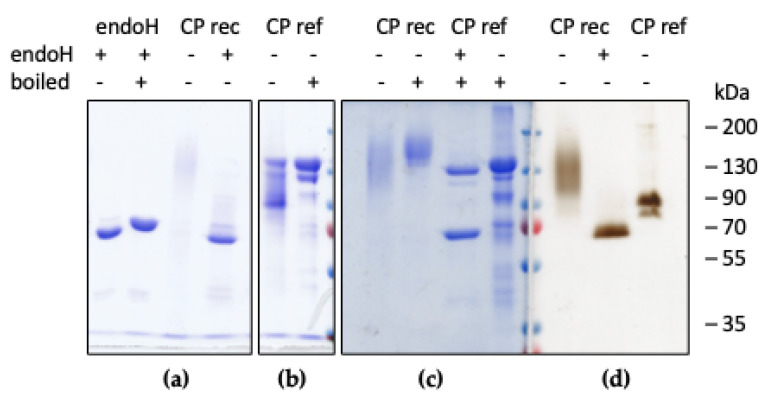
SDS-PAGE analysis of purified recombinant CP before and after treatment with endoH. Recombinant CP was incubated with EndoH in non-denaturing conditions. Samples of recombinant CP (CP rec, 1 μg in panel (**a**), 3 μg in panels (**b**–**d**)) and of plasma-derived CP (CP ref, 5 μg) were loaded with or without heat treatment as indicated. EndoH is shown in the first two lanes. Panels (**a**–**c**): Coomassie staining; panel (**d**): oxidase activity staining with oDA.

To further investigate the source of the higher apparent molecular weight observed for recombinant CP, deglycosylation with endoH was performed in non-denaturing conditions. SDS-PAGE analysis evidenced that after incubation with endoH the M_r_ of the protein decreased to about 120 kDa, as expected for single-chain human CP (Figure 3). Non-denaturing SDS-PAGE and staining for oxidase activity with oDA confirmed that recombinant CP is enzymatically active also after removal of oligosaccharide chains. The electrophoretic mobility of the oxidase-active bands for recombinant CP is different from that of plasma-derived CP due to the different glycan moieties of the two proteins. It should be recalled that plasma-derived CP is resistant to endoH due to the complex nature of the glycan chains [20], so treatment with EndoH was not performed in this case.

**Figure 4:**
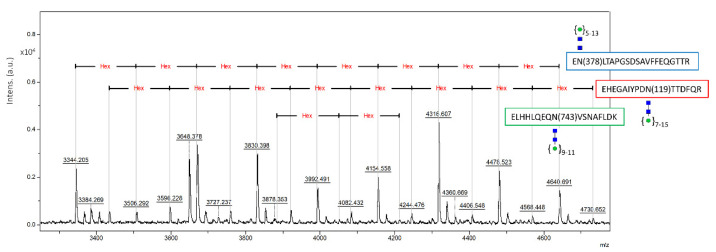
MALDI TOF spectrum of recombinant CP tryptic glycopeptides including their peptide backbones and glycans attached.

The amino acid sequence of human CP contains 7 potential N-glycosylation sites: Asn119, Asn339, Asn378 and Asn743 (which are modified by addition of complex oligosaccharide chains in plasma-derived CP) and Asn208, Asn569 and Asn907 (which are not occupied in the native protein) [31,36]. Glycan analysis has been performed on purified recombinant CP in order to determine glycan structure and occupancy at the different sites (Table 1 and Table 2, and Figure 4). Table 1 reports the main N-glycoforms (above 6% relative intensity) detected by MALDI TOF of permethylated N-glycans released from recombinant CP. Although SM5 is engineered to produce HexNAc2Hex5 structures, the two most abundant species were high-mannose HexNAc2Hex8 and HexNAc2Hex11. Off-target unexpected N-glycan structures have been detected in other proteins expressed in SM5, indicating that this is probably a common problem encountered with glycoengineered *P. pastoris* strains [37]. The dominating structures of these N-glycans were variable and appeared to be somewhat protein-dependent [37].

Determination of glycosite occupancy was performed by analysis of tryptic glycopeptides after their enrichment by HILIC SPE (Figure 4). The three N-glycosylation sites at Asn119, Asn378 and Asn743 were detected as occupied by high-mannose N-glycans predominantly of HexNAc2Hex7-8-9-11 structure. No glycopeptides bearing the Asn339 site were observed. N-glycan chains are missing at the Asn339 site in isoform II of human plasma-derived CP, that represents a minor form of the circulating protein [31], suggesting that this site may be less accessible for modification.

Extent of occupancy of the individual glycosylation sites was evaluated by their stable isotope labelling. In the process of enzymatic N-glycan cleavage by PNGase F hydrolase, asparagine residues with attached glycan are converted to aspartate. In the environment of ^18^O-water, created ^18^O-Asp indicates the presence of a glycosylation site that is observed by a mass shift in the subsequent proteomic analysis by nanoLC-MS/MS. In addition, enzymatic stable isotope labelling helps to distinguish the true glycosylation sites from a spontaneous Asn deamidation that may occur during the protein purification and storage. The presence of peptides with the original Asn indicates lack of glycosylation at the corresponding site. Based on this approach, glycosylation was confirmed at Asn119, Asn378, and Asn743. The glycosylation site at Asn339 was confirmed to be unoccupied by glycans as it was reported only as Asn (Table 2). High occupancy of 86% and 69% was found at Asn378 and Asn743, respectively. Instead, Asn119 appeared to be modified to a lower extent (38%). Thus, results obtained from analysis of occupancy of glycosylation sites suggest that three out of four glycosites, occupied in plasma-derived CP, were also present in analyzed recombinant CP. Original data from proteomic analysis of recombinant CP and its glycosylation sites is available as Supplementary Materials.

## 3. Materials and Methods

### 3.1. Yeast Strains

The glycoengineered *Pichia pastoris* SuperMan5 (*och1*Δ*1*, *GAP-mannosidaseHDEL*, *his4*Δ*1*) strain (SM5) was purchased from BioGrammatics (Carlsbad, CA, USA). The original *fet3*Δ strain derived from JC300 (*ade1*, *arg4*, *his4*) is described in [25]. Yeast cells were grown at 30 °C in YPD or in MD minimal medium with the appropriate auxotrophic supplements. Inactivation of the endogenous ferroxidase FET3 gene in SM5 was achieved by the split marker strategy. Briefly, a KanR marker expression cassette including KanR flanked by the GAP promoter and AOX1 terminator was amplified from pIB2KAN and it was inserted in pBS-FET3 [25] following digestion with *Bam*HI to replace 335 bp of FET3 coding sequence. The cassette was split by digestion with *Kpn*I/*Ssp*I and *Cla*I/*Bgl*II to produce two fragments, including about 600 bp of FET3 sequence and about 800 bp of KanR sequence each, with a 240 bp overlap. Electroporation of SM5 cells was performed with the two overlapping DNA fragments and cells were plated on YPD supplemented with 0.5 mg/mL G418. To screen for the inactivation of FET3, G418-resistant colonies were plated on MDH with or without the iron chelator BPS 20 μM, and after 48 h at 30 °C they were scored for lack of growth. Colonies with impaired growth in BPS were further screened by PCR on genomic DNA and lack of Fet3p oxidase activity to confirm inactivation of the FET3 gene. Genomic DNA was extracted according to [38] and PCR was carried out with the following primers: fet3_forward 5′-aggaattcatgtttgtattcgaacc-3′; kan_reverse 5′-gacctcgagttagaaaaactcatcgagcatc-3′ with the following conditions: 95 °C for 5 min followed by 25 cycles 95 °C 30 s, 58 °C 30 s, 72 °C 2 min 30 s and a final extension at 72 °C for 5 min.

### 3.2. Expression and Purification of Recombinant CP

The plasmid for constitutive expression of the secreted isoform of human CP is described in [20,25]. Site-directed mutagenesis by overlap extension PCR was performed to replace Arg481 and Lys887 with Gln in order to remove the two major sites responsible for susceptibility of CP to proteolysis [28]. A version of CP with the native signal peptide replaced with a hybrid Ost1/alfa-factor signal peptide described in [32] was produced by PCR and cloned in the same vector for constitutive expression as wild type CP. The pPICZ-pOst1-pro-alfaF(MUT1)-E2-Crimson from which the hybrid signal peptide was amplified was a gift from B.S. Glick (Addgene plasmid #117662). All plasmids were linearized with *Sal*I and electroporated in the SM5 *fet3*Δ strain. The presence of the CP coding sequence was confirmed by PCR on genomic DNA of selected His^+^ colonies.

Expression of secreted CP was performed in MD buffered with 50 mM potassium phosphate pH 6.8 supplemented with CuSO_4_ 50 μM and Fe(NH_4_)_2_(SO_4_)_2_ 50 μM. For small-scale analysis, 10-mL cultures were employed and culture supernatants were concentrated 30-fold with Millipore Ultra 30 K devices. One-litre cultures were used for purification of the protein. Supernatants from overnight cultures were filtered, diluted 1:3, brought to pH 7.1 and loaded onto DEAE-Sepharose (7.5 mL resin for 1 l culture medium) in 25 mM Mops buffer pH 7.0, supplemented with 30 mM NaCl. The resin was washed with the same buffer (10 column volumes) and CP was eluted with 25 mM Mops pH 7.0, containing 150–250 mM NaCl (4 column volumes). The CP-containing fraction was diluted 1:3 and loaded onto 1 mL Sepharose derivatized with chloroethylamine (AE-Sepharose) [33] in 25 mM Mops pH 7.0, containing 50 mM NaCl. The resin was washed with the same buffer (10 column volumes) and CP was eluted with 25 mM Mops pH 7.0, containing 250–500 mM NaCl (5 column volumes). CP-containing fractions were concentrated with Millipore Ultra 30K devices.

### 3.3. Characterization of Recombinant CP

Denaturing and non-denaturing SDS-PAGE and staining for oxidase activity with *o*-dianisidine were performed as described [20]. Western blot was performed with rabbit polyclonal anti-CP antibody (Dako) at 1:5000 dilution. Total protein content was evaluated by the Bradford assay (BioRad) using BSA as a standard. Deglycosylation with EndoH_f_ (NEB) was performed in non-denaturing conditions for 1 h at 37 °C.

Oxidase activity was determined with 1.58 mM *o*-dianisidine (oDA) in 0.5 mL 100 mM sodium acetate buffer pH 6.0 for 1 h at 37 °C. The reaction was stopped with 0.5 mL of 9 M H_2_SO_4_ and the amount of product was determined by absorbance at 540 nm. Ferroxidase activity of CP was assayed with 20–100 µM Fe(NH_4_)_2_(SO_4_)_2_ in 100 mM sodium acetate buffer pH 6.0 at 25 °C for 5–10 min. The amount of residual Fe^2+^ was measured by the addition of 1 mM ferrozine and determination of absorbance at 562 nm (ε_562_ 27,900 M^−1^ cm^−1^). Plasma-derived CP was purified by the single-step procedure described in [33]. The protein showed a 610/280 ratio > 0.04.

### 3.4. Glycan Analysis

#### 3.4.1. Analysis of N-Glycoprofile

Analysis of N-glycans was performed as described before [39]. Briefly, after the release of N-glycans with PNGase F (Roche), the sample was subjected to PGC chromatography. N-glycans were eluted, permethylated and analyzed by MALDI TOF in reflectron positive ion mode with the UltrafleXtreme MALDI mass spectrometer (Bruker Daltonics, Billerica, MA, USA).

#### 3.4.2. Analysis of N-Glycopeptides and Occupancy of Glycosylation Sites

Tryptic peptides bearing N-glycans were analyzed by MALDI TOF. Occupancy of glycosylation sites was determined by hydrolytic labelling of occupied Asn residues that were deamidated to ^18^O-Asp by nanoLC-MS/MS using Orbitrap Elite mass spectrometer (Thermo Fisher Scientific, Waltham, MA, USA). Full details of glycan and glycopeptide analysis are described in the Supplementary Materials.

## 4. Conclusions

If compared to the yields that can be obtained by the isolation of CP from human plasma where the concentration of the protein is about 300 mg/L, it is evident that there is no advantage in heterologous expression, where production levels are much lower. In fact, CP appears to be one of those cases where the natural source provides much more protein than the recombinant expression system. However, the availability of high-quality recombinant protein represents a significant advancement in the field of CP biology because it makes it feasible to design and produce site-specific mutants of CP. This breakthrough is crucial for studies aimed at unveiling the molecular basis of defective missense aceruloplasminemia mutants. Moreover, specifically designed mutants based on the available 3D structure of human CP will allow to define the details of the catalytic activity and of the molecular interaction of the ferroxidase with ferroportin.

On a different note, productivity would need to be greatly increased and, more importantly, the finding that HexNAc2Hex8 and HexNAc2Hex11 glycan chains are predominant in recombinant CP indicates that the glycan structure is probably unsuitable and further careful glycoengineering of the SM5 strain will be mandatory before any potential therapeutic use of the recombinant protein for enzyme replacement therapy of aceruloplasminemia patients can be envisaged.

## Figures and Tables

**Table 1:** Main N-glycoforms detected in recombinant human CP.

Structure	*m*/*z* (Permethylated)	Intensity	Relative Intensity (%)
HexNAc2Hex5	1579.971	764	6.1
HexNAc2Hex7	1988.437	1199	9.5
HexNAc2Hex8	2192.73	2211	17.6
HexNAc2Hex9	2397.024	1045	8.3
HexNAc2Hex10	2601.325	1443	11.5
HexNAc2Hex11	2805.58	1821	14.5
HexNAc2Hex12	3009.771	969	7.7

**Table 2:** Occupancy of glycosylation sites of CP determined by enzymatic ^18^O deamidation after PNGase F digest.

N-Glycosylation Site ^1^	Covered by Peptides	Ratio ^18^O-Asp/Asn after PNGase F ^3^	% of Glycosylation
Asn119 *	Yes	0.61012	38
Asn208	No ^2^	-	-
Asn339 *	Yes	ND	ND
Asn378 *	Yes	6.2394	86
Asn569	Yes	ND	ND
Asn743 *	Yes	2.2496	69
Asn907	Yes	ND	ND

^1^ Numbering is of the mature protein without the signal peptide; N-glycosylation sites occupied in the native CP are marked with an asterisk (*). ^2^ Neither peptides nor glycopeptides bearing Asn208 N-glycosylation site were observed. ^3^ Calculation of overall occupancy of respective glycosite based on peptide intensities bearing ^18^O-Asp and Asn.

## Data Availability

All data are reported in the manuscript and Supplementary Materials.

## Supplementary Materials

The following are available online at https://www.mdpi.com/article/10.3390/ijms22158228/s1, Supplementary methods with figures and data.
