# Supplementary material for: Production of Recombinant Human Ceruloplasmin: Improvements and Perspectives
Source: Int J Mol Sci. 2021 Jul 30;22(15):8228. doi: 10.3390/ijms22158228 (PMC8347646; doi:10.3390/ijms22158228)
Supplement: Supplementary file 1 [file ijms-22-08228-s001.zip › Suplementary Material.pdf]

International Journal of Molecular Sciences

MDPI

Supplementary Material

1. Methods

1.1. Analysis of N-glycans by MALDI TOF

90 µg protein sample was dried and restored in 10 mM TrisHCl pH 7.5 with 0.1 % SDS and incubated at 100°C for 10 min. Cooled sample was de-glycosylated o/n (37°C) with 1U of peptide-N-glycosidase (PNGase F; Roche Diagnostics GmbH, DE). After the release of N-glycans, the sample was subjected to PGC chromatography using Supelclean ENVI-Carb SPE system (Supelco/Sigma Aldrich, PA, USA). N-glycans were eluted with 60 % ACN with the addition of 0.1 % trifluoroacetic acid (TFA) (*v/v*), permethylated and analysed by MALDI TOF in reflectron positive ion mode with the UltrafleXtreme MALDI mass spectrometer equipped with a 1000 Hz Smartbeam™-II laser (Bruker Daltonics, MA, USA). A 20 mg/mL DHB solution in 30 % ACN that was supplemented with 0.1 % TFA and 1 mM NaOH was used as a matrix solution.

1.2. Analysis of N-Glycopeptides by MALDI TOF

15 µg of protein was dissolved in 50 mM Tris pH 7.8. Reduction and alkylation of disulphide bonds was performed by 5 mM DTT (60°C, 30 mins) and 15 mM IAA (RT, dark, 20 mins) with subsequent quenching by 5 mM DTT. For proteolytic digestion, sequencing grade modified trypsin (Promega Corp., WI, USA) was added in the ratio 1:20 and reaction was incubated overnight at 37°C.

N-glycopeptides were enriched by HILIC SPE. Briefly, sample was dissolved in 83 % ACN and the home-made HILIC column, filled with 3 mm of cotton in a 10 µl pipette tip, was washed with H_2O and conditioned with 83 % ACN. After loading the sample, column was washed by 83 % ACN with 0.1 % TFA and enriched glycopeptides were eluted by 10 µl H_2O. For MALDI TOF analysis, 1 µl of eluted glycopeptides was premixed onto the target plate with 1 µl of matrix, consisting of 20 mg/ml Na-DHB in 30 % ACN and 0.1 %TFA. Sample was analysed in reflectron positive ion mode by UltrafleXtreme II mass spectrometer (Bruker, MA, USA). Theoretical masses of peptides bearing N-glycosylation sites were calculated by PeptideMass tool from Expasy bioinformatics portal and attached glycan structures were calculated manually.

1.3. Analysis of glycosylation sites by LC-ESI-Orbitrap Elite™

15 µg of protein sample was modified by DTT/IAA in 50 mM Tris pH 7.8 as described above (1.2.), and digested with trypsin overnight. Trypsin was inactivated (0.5 % TFA) and peptides were purified by C18 SPE and eluted in 70 % ACN. Dried peptides were restored in ^{18}O-water and 50 mM AmBic. Half of the sample was treated with peptide-N-glycosidase (PNGase F; Roche Diagnostics GmbH, DE), the other half served as negative control for monitoring the spontaneous peptide deamidation. The samples were incubated at 37°C o/n. The reaction was terminated by the addition of 0.1 % TFA and the peptides were analysed by nanoLC-MS/MS using Orbitrap Elite™ mass spectrometer (Thermo Fisher Scientific Inc., MA, USA). The data were processed by MaxQuant software package with carbamidomethylation (C) as permanent and oxidation (M) and ^{18}O-deamidation (N) as variable modifications.

2. Figures and Tables

Figure S1

MALDI TOF spectrum with assigned structures of peptide-N-glycosidase released and subsequently permethylated N-glycans from human recombinant CP protein. High mannose N-glycans, from HexNAc2Hex5 (*m/z* 1580, $[M + Na]^+$) to HexNAc2Hex28 (*m/z* 6274.1, $[M + Na]^+$) were observed.

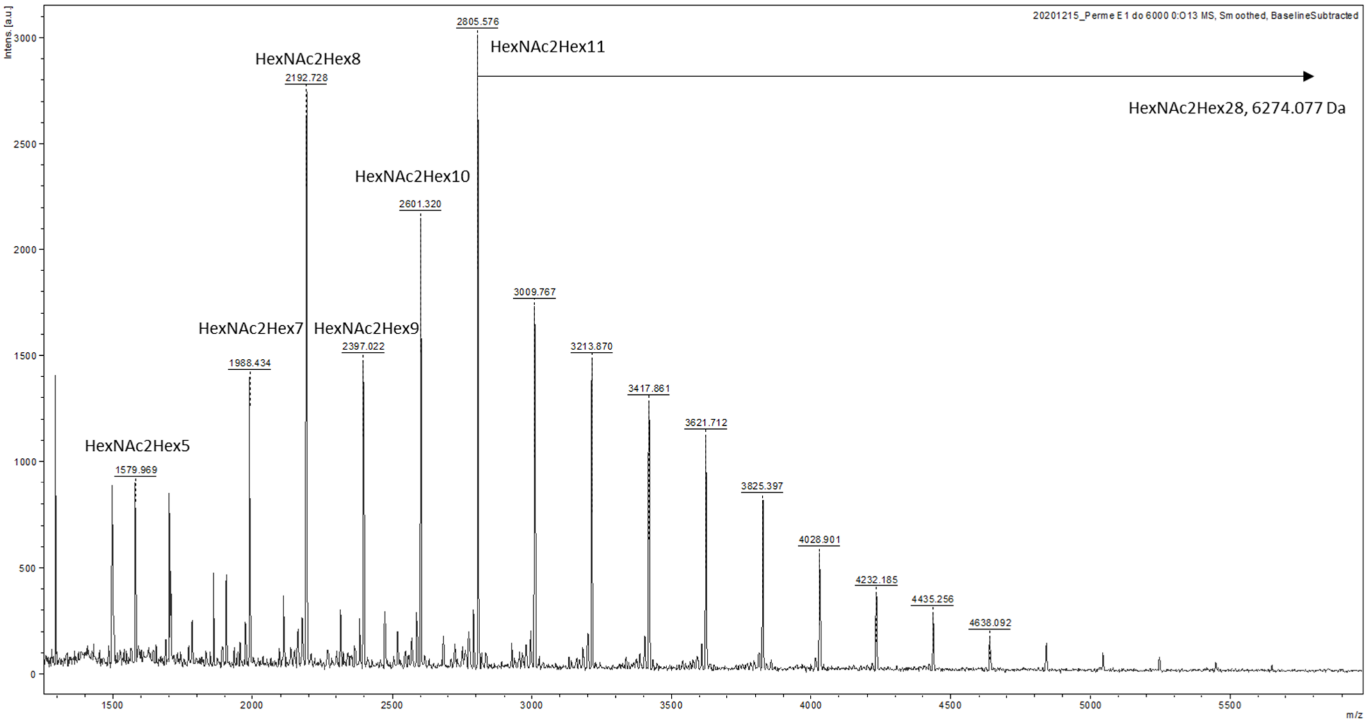

Table S1

List of N-glycans observed in the human recombinant CP. Relative signal intensities quantifications of high mannose N-glycans revealed two most abundant N-glycan structures HexNAc2Hex11 (*m/z* 2805.6, $[M + Na]^+$) and HexNAc2Hex8 (*m/z* 2192.7, $[M + Na]^+$), highlighted by red color.

m/z (Permethylated, $[M + Na]^+$)	Structure	Intensity	Relative intensity (%)
1579.971	HexNAc2Hex5	764	6.1
1784.178	HexNAc2Hex6	236	1.9
1988.437	HexNAc2Hex7	1199	9.5
2192.73	HexNAc2Hex8	2211	17.6
2397.024	HexNAc2Hex9	1045	8.3
2601.325	HexNAc2Hex10	1443	11.5
2805.58	HexNAc2Hex11	1821	14.5
3009.771	HexNAc2Hex12	969	7.7
3213.874	HexNAc2Hex13	716	5.7
3417.866	HexNAc2Hex14	594	4.7
3621.717	HexNAc2Hex15	472	3.8
3825.402	HexNAc2Hex16	329	2.6
4028.907	HexNAc2Hex17	209	1.7
4232.19	HexNAc2Hex18	134	1.1
4435.26	HexNAc2Hex19	88.3	0.7
4638.09	HexNAc2Hex20	50.2	0.4
4840.615	HexNAc2Hex21	33.9	0.3
5043.88	HexNAc2Hex22	67.5	0.5
5246.959	HexNAc2Hex23	65.9	0.5
5450.515	HexNAc2Hex24	46.3	0.4
5650.067	HexNAc2Hex25	39.5	0.3
5850.996	HexNAc2Hex26	22.9	0.2

Figure S2

MALDI TOF spectrum of human recombinant CP tryptic peptides and glycopeptides. Series of determined glycopeptides is marked by red rectangle and started at *m*/*z* 3182 [M + H]$^+$.

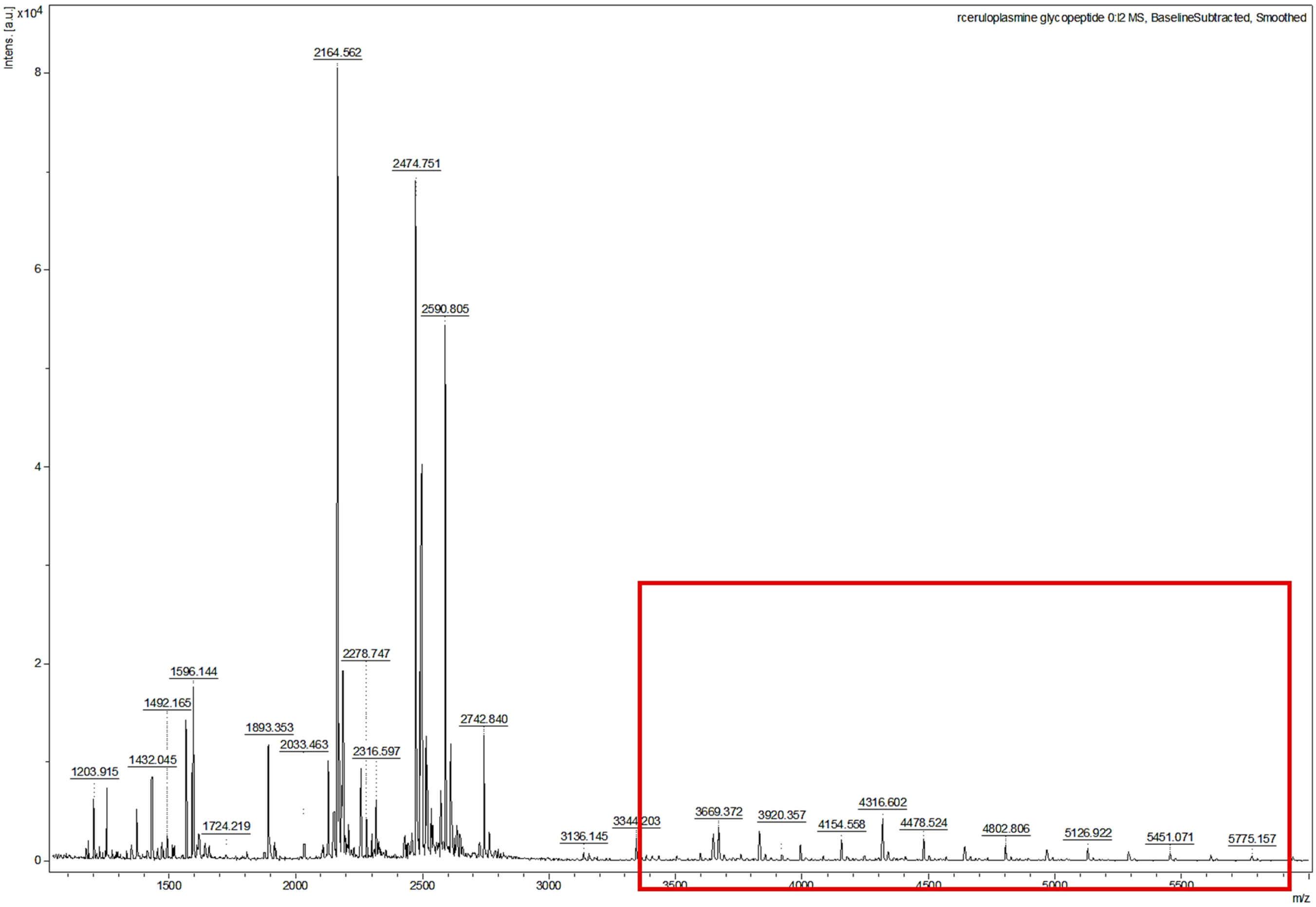

Table S2

Summary of identified human recombinant CP tryptic glycopeptides including their peptide backbones and attached glycans. Highlighted (Red) are the most abundant glycopeptide forms. No glycopeptides bearing N339 glycosylation site were observed.

N-glycosylation site (UniProt)	Peptide backbone (theoretical MW, average)	N-glycan structure	m/z values $[M + H]^+$
N378	ENLTAPGSDSAVFFEQGTTR (MW 2127.25 Da)	HexNAc2Hex4-6,<br>HexNAc2Hex7,<br>HexNAc2Hex8,<br>HexNAc2Hex9,<br>HexNAc2Hex10,<br>HexNAc2Hex11,<br>to HexNAc2Hex20	3182 - 3506,<br>3669,<br>3830,<br>3992,<br>4154,<br>4316,<br>to 5775
N119	EHEGAIYPDNTTDFQR (MW 1892.96 Da)	HexNAc2Hex7,<br>HexNAc2Hex8,<br>HexNAc2Hex9,<br>HexNAc2Hex10,<br>to HexNAc2Hex15	3434,<br>3596,<br>3758,<br>3920,<br>to 4730
N743	ELHHLQEQNVSNAFLDK (MW 2022.20 Da)	HexNAc2Hex8,<br>HexNAc2Hex9,<br>HexNAc2Hex10,<br>HexNAc2Hex11,<br>to HexNAc2Hex20	3726,<br>3888,<br>4049,<br>4211,<br>to 5670
N339	not observed		
